# Ginsenoside Rg1 and platelet-rich fibrin enhance human breast adipose-derived stem cells function for soft tissue regeneration

**DOI:** 10.18632/oncotarget.9360

**Published:** 2016-05-14

**Authors:** Fang-Tian Xu, Zhi-Jie Liang, Hong-Mian Li, Qi-Liu Peng, Min-Hong Huang, De-Quan Li, Yi-Dan Liang, Gang-Yi Chi, De-Hui Li, Bing-Chao Yu, Ji-Rong Huang

**Affiliations:** ^1^ Department of Orthopedics, The First Affiliated Hospital of Gannan Medical University, Ganzhou 341000, China; ^2^ Department of Hepatobiliary and Gland Surgery, The Fifth Affiliated Hospital of Guangxi Medical University & The First People's Hospital of Nanning, Nanning 530022, China; ^3^ Department of Plastic and Aesthetic Surgery, The Fifth Affiliated Hospital of Guangxi Medical University & The First People's Hospital of Nanning, Nanning 530022, China; ^4^ Central Laboratory of Medical Science, The Fifth Affiliated Hospital of Guangxi Medical University & The First People's Hospital of Nanning, Nanning 530022, China; ^5^ Department of Breast Surgery, The Affiliated Tumor Hospital of Guangxi Medical University, Nanning 530021, China; ^6^ Department of Burns and Plastic Surgery, The First Affiliated Hospital of Guangxi Medical University, Nanning 530021, China

**Keywords:** human breast adipose-derived stem cells, collagen type I sponge scaffolds, ginsenoside Rg1, platelet rich fibrin, soft tissue regeneration

## Abstract

Adipose-derived stem cells (ASCs) can be used to repair soft tissue defects, wounds, burns, and scars and to regenerate various damaged tissues. The cell differentiation capacity of ASCs is crucial for engineered adipose tissue regeneration in reconstructive and plastic surgery. We previously reported that ginsenoside Rg1 (G-Rg1 or Rg1) promotes proliferation and differentiation of ASCs *in vitro* and *in vivio*. Here we show that both G-Rg1 and platelet-rich fibrin (PRF) improve the proliferation, differentiation, and soft tissue regeneration capacity of human breast adipose-derived stem cells (HBASCs) on collagen type I sponge scaffolds *in vitro* and *in vivo*. Three months after transplantation, tissue wet weight, adipocyte number, intracellular lipid, microvessel density, and gene and protein expression of VEGF, HIF-1α, and PPARγ were higher in both G-Rg1- and PRF-treated HBASCs than in control grafts. More extensive new adipose tissue formation was evident after treatment with G-Rg1 or PRF. In summary, G-Rg1 and/or PRF co-administration improves the function of HBASCs for soft tissue regeneration engineering.

## INTRODUCTION

Engineered adipose tissue is an attractive substitute for reconstruction or augmentation of soft tissue defects in reconstructive, plastic, or aesthetic surgery. Unfortunately, most current autograft techniques fail to produce long-term satisfactory replacement, in part due to the fragility of adipocytes and the lack of appropriate vascularization after grafting. Trauma, tumor resection, and congenital or acquired anomalies are the main causes justifying the need for adipose substitutes in reconstructive surgery, and tissue engineering strategies are a promising alternative therapeutic approach to address the low predictability of autologous fat transplantation.

Previous studies have proven that an ideal scaffold could promote the tissue regeneration process, an important factor in improving the cosmetic result [1–4]. Stem cells are commonly seeded on 3D scaffolds where they proliferate, differentiate, and secrete specific ECM molecules which promote additional scaffold formation, cell adhesion, and further proliferation both *in vitro* and *in vivo*. Scaffolds induce angiogenesis pathways leading to vascularization in neogenetic tissue *in vitro*. A three-dimensional, porous collagen sponge is similar to the extracellular matrix, compatible with the human body, and an ideal microenvironment for adhesion, spread, and proliferation of ASCs or MG-63 cells [5–6]. Excellent biocompatibility which can be affected by many factors including drugs, microenvironment, or endocrine changes is a critical requirement for scaffolds in adipose tissue engineering [7–9].

Ginsenoside Rg1, the active component of ginseng, possesses various therapeutic actions [10–13]. Our previous work indicated that ginsenoside Rg1 promotes proliferation and neural differentiation of human ASCs *in vitro*, suggesting a potential use for human ASCs in neural regenerative medicine [14]. Additionally, it was shown that platelet-rich fibrin (PRF), a concentrate of autologous platelets on a fibrin membrane without added external factors, has a high potential for tissue repair. Advanced platelet-rich fibrin (A-PRF) influences bone and soft tissue regeneration through the presence of monocytes/macrophages and their respective growth factors (PRF was centrifuged at 2700 rpm for 12 min from 10 ml of whole blood without anticoagulant whereas A-PRF was centrifuged at 1,500rpm for 14 min.) [15–17] In this study, we selected G-Rg1 and PRF to pre-treat human breast adipose-derived stem cells (HBASCs) seeded on three-dimensional porous collagen type I sponge scaffolds. We then detected cell survival, proliferation, differentiation, angiogenesis, and adipose tissue regeneration *in vitro* and *in vivo*.

## RESULTS

### Characterization and multipotency of HBASCs

**Figure 1:**
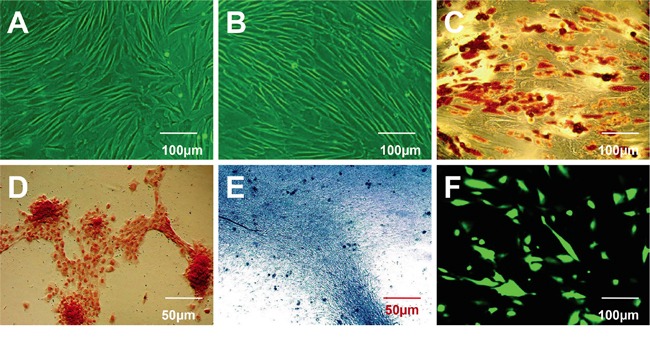
Characterization of human breast adipose-derived stem cells (HBASCs) prior to and following induction of multilineage differentiation **A.** Morphological characterization of HBASCs at passage 0. **B.** Morphological characterization of HBASCs at passage 3 were: **C.** positive for oil red O staining following adipogenic induction for 2 weeks; **D.** positive for alizarin red staining following osteogenic induction for 3 weeks; **E.** positive for alcian blue staining following chondrogenic induction for 2 weeks; and **F.** GFP+. Scale bars: 50 μm (D, E); 100 μm (A, B, C, F).

Following initial isolation and expansion, homogeneous HBASCs that grew in a monolayer with spindle-shaped morphology were observed following one to two weeks culture (Figure 1A). Cells were harvested at 80-90% confluence and passaged at a ratio of 1:3. Isolated, subconfluent HBASCs at 3^rd^ passage (Figure 1B) were cultured for 1-2 weeks with adipogenic, osteogenic, and chondrogenic induction. Lineage-specific cell morphology was observed following 2, 3, and 2 weeks of inductive culturing for adipocytes, osteoblasts, and chondrocytes, respectively. Positive Oil Red O, alizarin red, and alcian blue staining typically indicate adipocytes, osteoblasts, or chondrocytes, respectively. The results confirm that the HBASCs differentiated into adipocytes, osteoblasts, and chondrocytes following relevant inductive culturing, validating their multipotency (Figure 1C, 1D, 1E). Green nuclei were observed in HBASCs when the cells were labeled with GFP (Figure 1F).

### Influence of G-Rg1 and PRF on the proliferation of scaffold-cultured HBASCs

**Figure 2:**
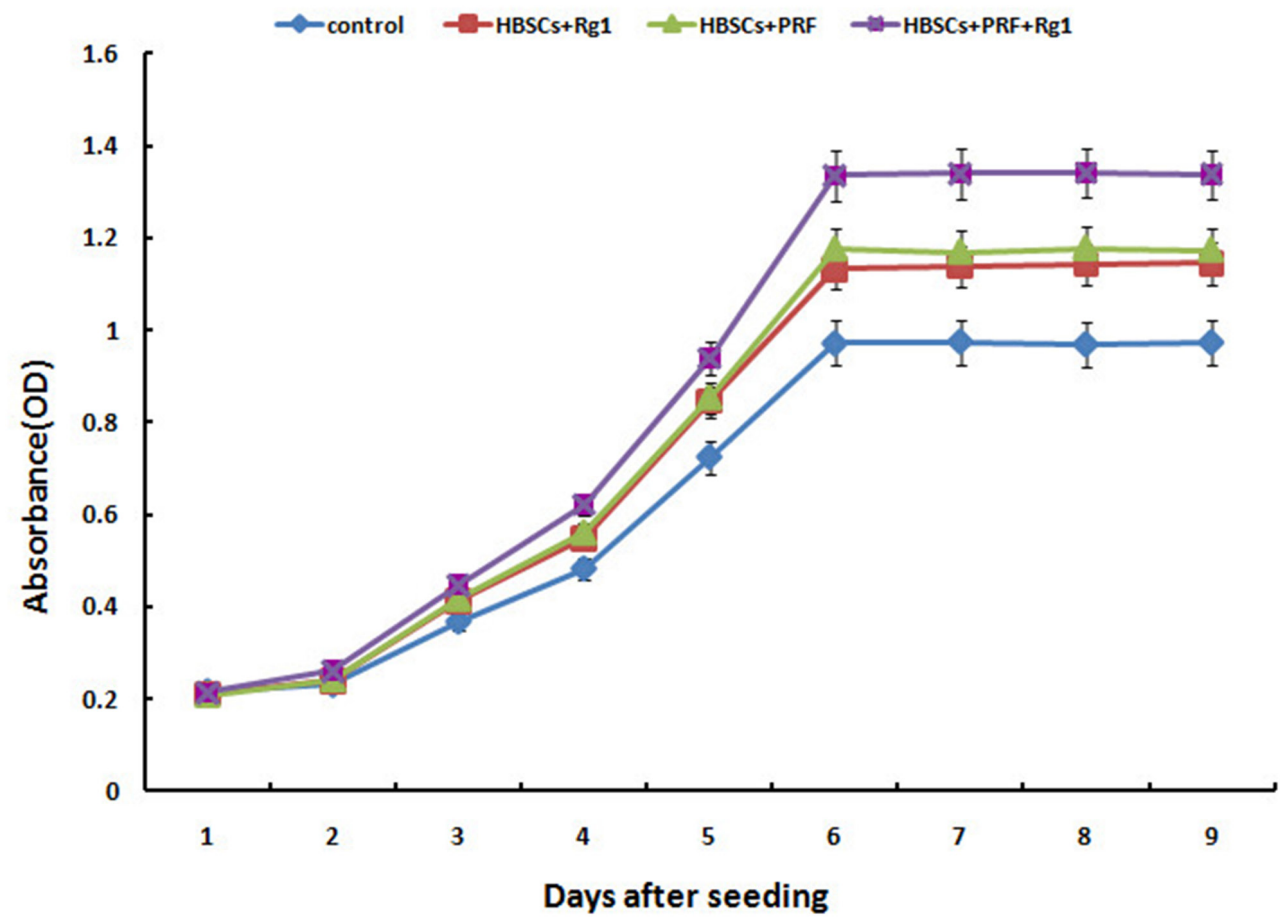
Results of the cell proliferation assay using the CCK-8 test Group B (10μg/ml Rg1), group C (10 mg/mlPRF), and group D (10μg/ml Rg1+10 mg/mlPRF) displayed higher absorbance than the control group (group A) at every time point starting from day 3 of the study. The group D displayed higher absorbance than groups B and C at every time point starting from day 3. Results are the mean ± SD, n = 6, *p* < 0.01, as assessed using ANOVA.

During the process of HBASCs culture with or without G-Rg1 and PRF, CCK-8 tests were performed on the 4 experimental groups. Ginsenoside Rg1 or PRF promoted the HBASCs cell proliferation during the logarithmic growth phase. Beginning 3 d after treatment, the proliferation rate of the ginsenoside Rg1 (group B) and PRF (group C) was higher than that of the control group (group A), and the combination of ginsenoside Rg1 with PRF (group D) augmented this effect (*p*< 0.001, Figure 2).

### Attachment of HBASCs on scaffolds

**Figure 3:**
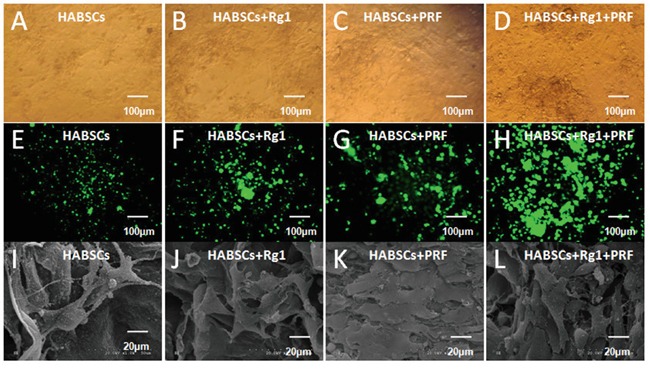
The morphology and status of HBASCs after co-culture with COL-S **A–H.** HBASCs under phase contrast and fluorescence microscopy after co-culture 3 days in 4 groups. **I–L.** HBASCs under SEM after co-culture 7 days in 4 groups.

After 3 days of culture on the scaffold, most HBASCs showed spindle-shape or polygon-shape morphology and adhered to the scaffold surface. Fluorescence imaging showed that almost all HBASCs of the 4 groups were viable. In addition, the density of live cells under phase contrast and fluorescence microscopy (green) was consistent with the results of the CCK-8 assays (Figure 3A-3H). Furthermore, to determine whether cells could attach to the scaffold, the morphology of HBASCs was observed by SEM after 7 days of culture. After 7 days co-culture, the number of HBASCs increased markedly and migrated into the pore of the scaffolds. The cells extended pseudopodia and adhered to the surface or pores. The cells aggregated, adhered, and grew on the scaffold, and secreted extracellular matrix. In the control group, attached cells were less frequently observed on the scaffold alone but were more frequently observed on the scaffold combined with Rg1, PRF, or both. These results are consistent with the findings for proliferation of CCK-8 assays (Figure 3I-3L) and show that collagen scaffolds are useful for cell attachment and growth.

### Macroscopic findings and histopathological assessment of neogenetic tissue

**Figure 4:**
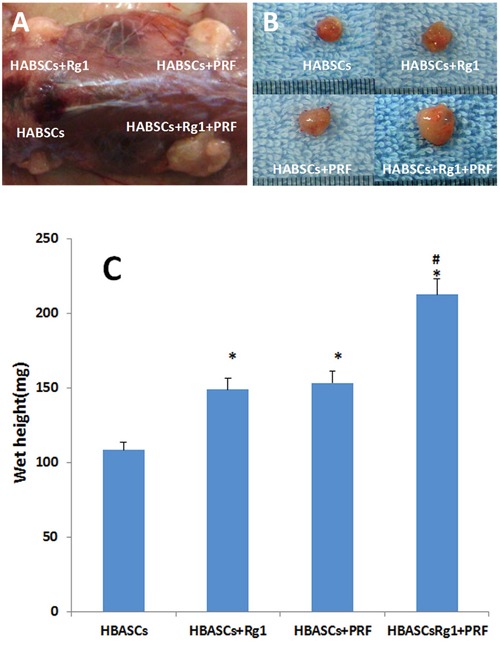
Macroscopic findings and wet weight of the transplants in 4 groups **A, B.** Macroscopic findings of the neogenetic tissue formation, excised from nude mice in 4 groups, **C.** The wet weight of the neogenetic tissue were measured and compared between the 4 groups. Results are the mean ± SD, n = 20, *p <0.01, #*p* <0.01, as assessed using ANOVA.

No animals died over the 3 months after transplantation. Neogenetic tissues formed and were excised as shown in Figure 4A and 4B. The transplant wet weight was measured by electronic balance, and the data of groups A to D are shown in Table 1. The differences of group D vs group A, group D vs group B, group D vs group C, group C vs group A, and group B vs group A were all statistically significant (Figure 4C, *p <0.01, #*p* <0.01). H&E staining showed that the neogenetic tissues were mature adipose tissue in all four groups. Group D consisted predominantly of mature adipose tissue and had less fibrosis compared to the other 3 groups; groups B and C also consisted predominantly of mature adipose tissue and had less fibrosis than group A; and group A consisted partially of mature adipose tissue and had more fibrosis than the other 3 groups. Moreover, slight inflammation and degradation of the three-dimensional porous collagen sponge were observed 3 months after implantation in nude mice (Figure 5A1-5D1).

**Table 1:** The wet tissue weight in each group (mg, Mean ± SD, n=20)

group	Control (group A)	HBASCs+Rg1 (group B)	HBASCs+PRF (group C)	HBASCs+Rg1+PRF (group D)
Wet weight	108.4±11.2	^*^148.91±15.1	^*^153.3±16.5	^#^^*^212.6±23. 3

* VS Control, *p*<0.01

# VS group B and group C, *p*<0.01

**Figure 5:**
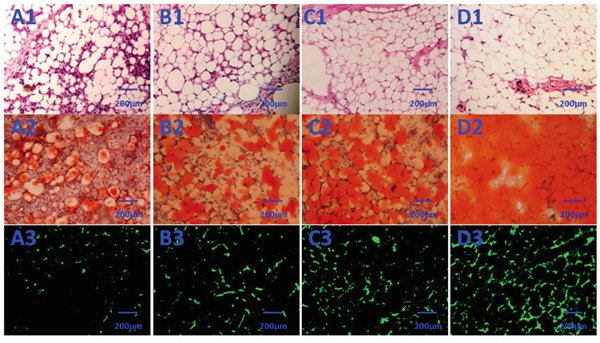
Histological evaluation of the neogenetic tissue after 3 months (×200) **A1–D1:** (D1) Group D consisted predominantly of mature adipose tissue and had significantly less fibrosis than the other 3 groups; C1 and B1: groups B and C also consisted predominantly of mature adipose tissue and had significantly less fibrosis than group A. A1: group A consisted partially of mature adipose tissue and had more fibrosis than the other 3 groups. **A2–D2:** In contrast to control (A2), the transplanted HBASCs treated with Rg1 or PRF (B2 and C2) contained more large Oil Red O-positive lipid droplets within their cytoplasm, and HBASCs combination with both Rg1 & PRF (D2) augments this effect. **A3–D3:** GFP+ cells were detected in the neogenetic mature adipose tissue, indicating that these mature adipocytes differentiated from GFP-labeled HBASCs.

### Origin of neogenetic adipose tissue and quantitative measurement of adipogenesis

**Figure 6:**
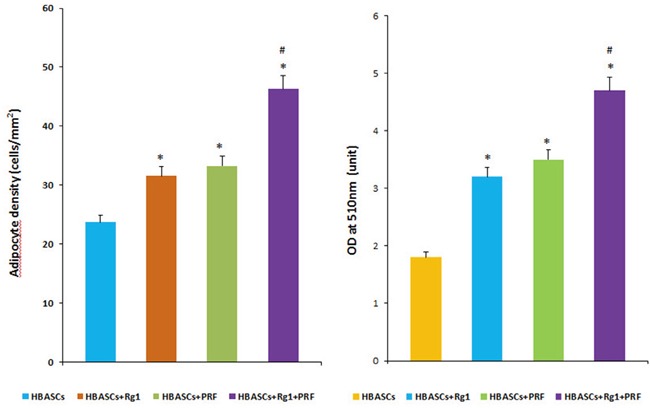
Quantitative measurement of adipogenesis in 4 groups The number of adipocytes and amount of intracellular lipid were higher in groups B and C than in the control group, and HBASCs treatment with both Rg1 & PRF (group D) augments this effect (Figure 6, *p <0.01; #*p* <0.01).

GFP+ cells were found in the neogenetic mature adipose tissue, which indicates that this mature adipocyte had differentiated from GFP-labeled HBASCs (Figure 5A3-5D3). Adipogenesis and lipid vacuole formation in the neogenetic mature adipose tissue sections were studied by Oil Red O staining, which showed the different adipogenic potentials of HBASCs in the four groups. In contrast to control, the transplanted HBASCs combined with Rg1 or PRF (group B or group C) contained more large Oil Red O-positive lipid droplets within their cytoplasm, and HBASCs combination with both Rg1 & PRF (group D) augments this effect (Figure 5A2-5D2). The number of adipocytes and intracellular lipid content were higher in group B or group C than in the control group; HBASCs combination with both Rg1 & PRF (group D) augments this effect (Figure 6, *p <0.01, #*p* <0.01).

### Micro vessel density of neogenetic adipose tissue in each group

**Figure 7:**
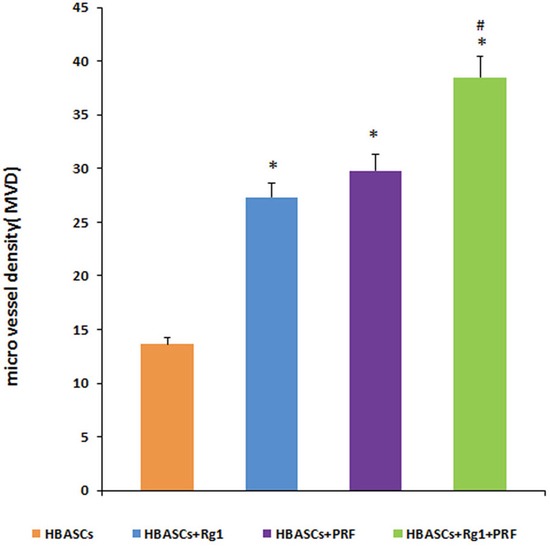
Micro vessel density (MVD) in the neogenetic tissue Histological evaluation of 10 fields per section taken from the center of the mature adipose tissue showed that MVD was much higher in group D than in the other three transplant groups. These differences were statistically significant, as determined by paired T-test. * *p* <0.01; # *p* <0.01.

Histological evaluation of 10 fields per section taken from the center of the neogenetic fat tissue allowed us to determine micro vessel density (MVD), an index of neovascularization. MVD was much higher in group D than in the other three groups. The HBASCs transplants mixed with Rg1 or PRF also had a reasonably good MVD. In contrast, few micro vessels could be detected in the controls. (Figure 5A1-5D1 and Figure 7, *p <0.01, #*p* <0.01).

### PPARγ, HIF-1α, and VEGF protein & gene expression in neogenetic adipose tissue

**Figure 8:**
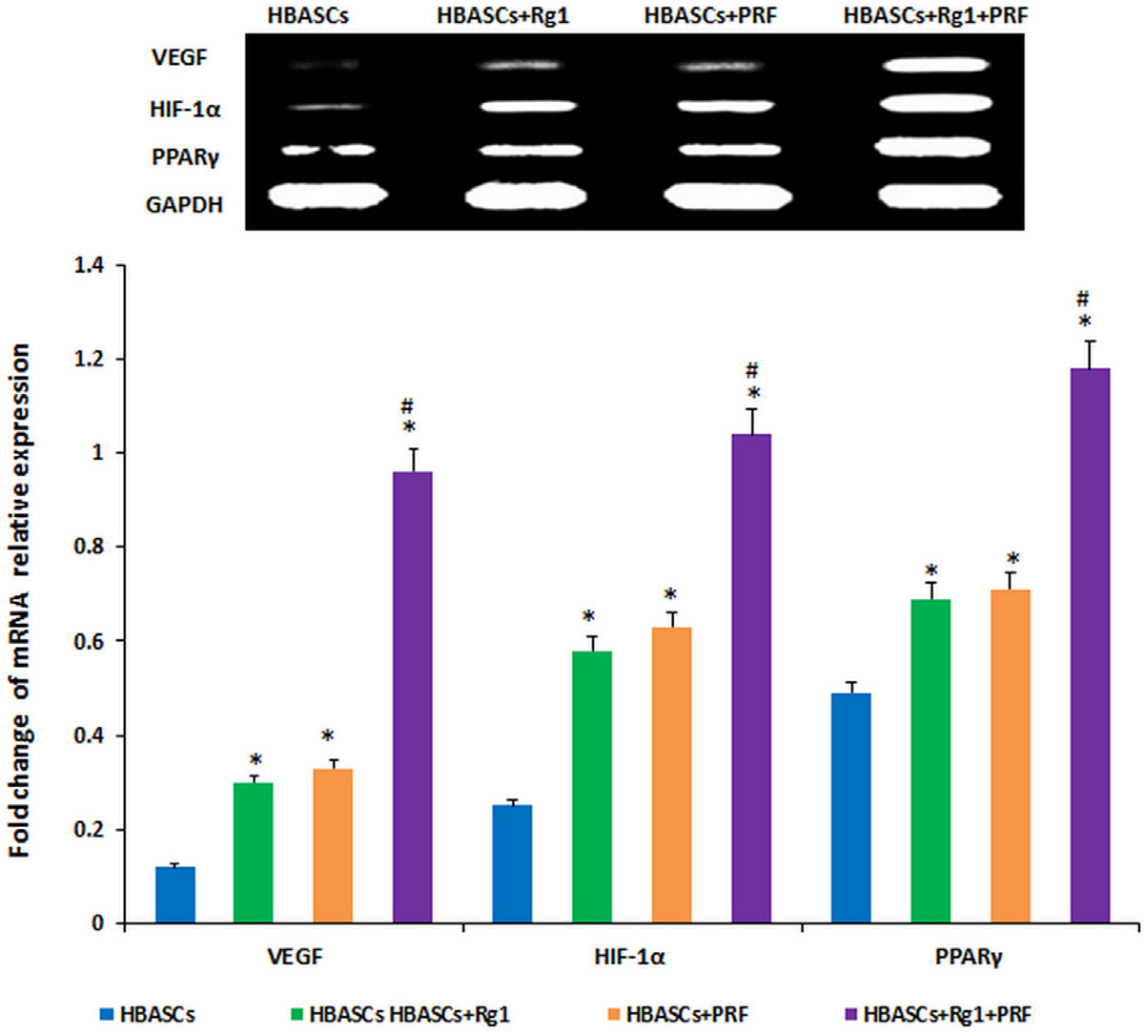
mRNA expression of PPARγ, HIF-1α, and VEGF were much higher in neogenetic adipose tissue of group D than in the other three groups, and had a reasonably higher expression in the single treated groups In contrast, few mRNAs were upregulated in the control group,* *p* <0.01; # *p* <0.01

**Figure 9:**
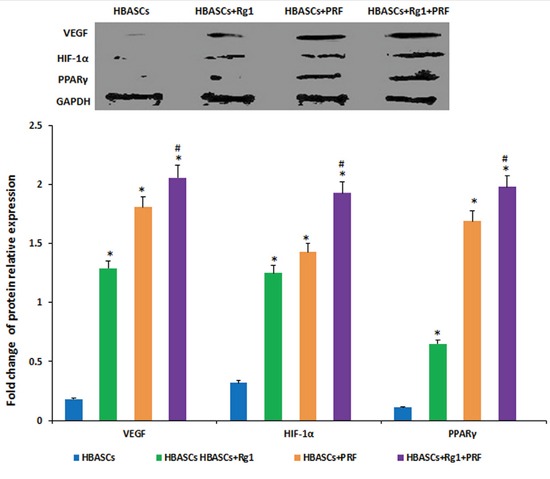
The protein expression of PPARγ, HIF-1α, and VEGF were much higher in neogenetic adipose tissue of group D than in the other three groups, and had a reasonably higher expression in the single treated groups In contrast, few mRNAs were upregulated in the control group, * *p* <0.01; # *p* <0.01

Three months after implantation, mRNA relative expression was measured by real-time quantitative PCR. The mRNA expression of PPARγ, HIF-1α, and VEGF were much higher in neogenetic adipose tissue of group D than in the other three groups, and the three genes had a reasonably higher expression in the transplants of HBASCs single mixed with Rg1(group B) or PRF(group C). The protein levels of PPARγ, HIF-1α, and VEGF were similar to the mRNA expression in all groups (Figures 8 and 9, *p<0.01, #*p* <0.01).

## DISCUSSION

Current reconstruction procedures, especially after trauma, congenital deformity, and oncological surgery, that transfer autologous soft tissue grafts have serious limitations. Multipotent adipose-derived stem cells (ASCs) are an extremely promising avenue for improving soft tissue defect repair and reconstruction. ASCs could induce either generation of soft tissue with a minor loss of adipose tissue at the donor site or increase the survival and durability of other grafts. Bioscaffolds serve as excellent framework for ASCs adhesion, growth, and proliferation, helping to maintain ideal graft morphology [22]. Alharbi Z et, al reported that freshly isolated uncultured ASCs can be safely seeded onto collagen and elastin matrices for *ex vivo* cellular enrichment after liposuction [23]. In this study, we chose collagen type I sponge as scaffold for HBASCs in order to evaluate its value for soft tissue regeneration.

Numerous previous studies have identified adipose-derived stem cells (ASCs) as a potential stem cell population with proliferation and differentiation ability similar to bone marrow-derived stem cells (BMSCs) [24–26]. ASCs are more easily isolated and can differentiate toward the osteogenic, chondrogenic, adipogenic, myogenic, neurogenic and angiogenic lineages [27–32].

Our previous studies proved platelet rich plasma promotes the proliferation of HBASCs and increases their eventual conversion rate into mammary gland like epithelial cells [33], and that HBASCs and CXCR4 transfection can enhance the survival and quality of transplanted free fat tissues [19]. Yang reported that HBASCs have the potential to transdifferentiate into human mammary epithelial-like cells when co-cultured with breast epithelial cells (HBL-100 cell line) [34]. Das indicated that HBASCs induce penile angiogenesis and neural regeneration without systemic inflammation in diabetic mice [35]. In our experiment, HBASCs were the transplant seed cells, and their proliferation and biological activity are improved by G-Rg1 or PRF.

Vascularization of the cell-scaffold transplant can be increased by many cytokines, including HIF-1α, VEGF, and PDGF. Gao reported that ASCs overexpressing HIF-1α accelerate neovascularization in ischemic diabetic skin flap [36], and Wang suggested that HIF-1α gene-modified ASCs accelerate the recovery of acute renal injury *in vitro* [37]. Moreover, hypoxia increases ASCs proliferation through HIF-1α activation [38], and VEGF stimulates ASCs growth VEGF [39]. Both Hye and Gehmert found that PDGF pretreatment of ASCs before transplantation increased neovascularization [40–41]. We found that HIF-1α and VEGF protein and mRNA levels were increased by treatment with G-Rg1, PRF. PRF is a new generation of platelet concentrate first described by Choukroun. It influences bone, cartilage and soft tissue regeneration through the presence of monocytes/macrophages and their growth factors including VEGF, PDGF, TGF-β, EGF, FGF, and IGF [42–45]. PRF promotes proliferation and differentiation of human oral bone mesenchymal stem cells *in vitro* [45–47].

We speculate that the paracrine activity of cytokines secreted by HBASCs is increased by G-Rg1. The higher level of cytokines more effectively promotes HBASCs proliferation and adipogenesis early after cell seeding, forming a positive feedback loop. The molecular mechanism of that loop is an interesting subject for further research.

We found that mixing G-Rg1 or/and PRF with autologous HBASCs increased their MVD density and viability. G-Rg1 and PRF improved the biocompatibility of COL-S and HBASCs, and increased HBASCs growth, proliferation, adhesion, survival, and adipogenic differentiation *in vitro* or *in vivo*. Combined application of ginsenoside Rg1 and PRF augmented these effects. At the same time, slight inflammation and degradation of the three-dimensional porous collagen sponge were observed 3 months after implantation in nude mice. Our data suggest that Rg1 and PRF would be ideal adjuvant to apply for cell assisted lipotransfer improving the biocompatibility of 3D porous collagen type I scaffolds. Three months after cells-scaffolds transplantation, there were neogenetic mature adipose tissue formation arising from the HBASCs. Tissue wet weight, the number of adipocytes, and intracellular lipid content were higher in HBASCs treated with G-Rg1 or PRF treated group than in the control group, and combination of the two (group D) augments this effect. While the molecular mechanism by which G-Rg1 and PRF enhance the survival of the HBASCs-scaffold transplants remains unclear, our data support the following possibilities. First, we found that the mRNA and protein expression of HIF-1α and VEGF were highest in neogenetic adipose tissue of the Rg-1/PRF combined treatment group; the single treatment groups also had a reasonably higher expression. In contrast, few mRNAs or proteins were upregulated in the control groups. VEGF and HIF-1α may promote the vasculogenesis that enhances the survival of the transplant. Second, we detected adipogenic differentiation of the GFP-labeled HBASCs in the neogenetic adipose tissue, which indicates that all of the adipocytes in the tissue were derived from the exogenous HBASCs. Moreover, the mRNA and protein expression of PPARγ were highest in neogenetic adipose tissue of the Rg-1/PRF combined treatment group; the single treatment groups also had a reasonably higher expression. Third, since the VEGF and HIF-1α enchanced HBASCs-scaffold transplant survival, it is possible that the hypoxic conditions in which the HBASCs with G-Rg1 and PRF found themselves in early after transplantation induced them to release soluble angiogenic factors such as VEGF and HIF-1α which promoted early transplant neovascularization and enhanced HBASCs-scaffold transplant survival. However, the detailed regulatory mechanism of G-Rg1 or PRF remains to be further explored.

## MATERIALS AND METHODS

### Patient consent and ethical approval

Animal experiments were carried out in strict accordance with the recommendations in “The Guide for the Care and Use of Laboratory Animals of the State Committee of Science and Technology of the People's Republic of China (2015)”. Experimental protocols were reviewed and approved by the Research Ethics Committee of the Guangxi Medical University (on 02/28/2015) and Gannan Medical University (Permit Number: 2015048).

### Scaffold preparation

**Figure 10:**
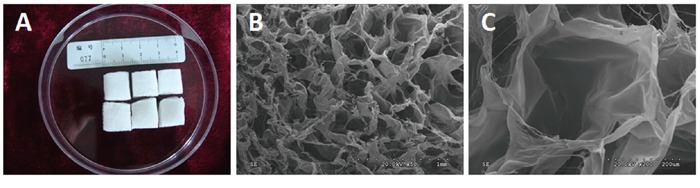
Macroscopic morphology and ultrastructure of the three-dimensional porous collagen type I sponge scaffolds **A.** Macroscopic morphology; **B.** SEM×50; **C.** SEM×200.

Three-dimensional porous collagen type I sponge scaffolds were supplied by Guangzhou Chuang'er Biotechnological Limited Company, China. They were stored in a dry cabinet at 17% relative humidity and UV sterilized prior to use. The collagen sponge was flexible and highly porous, with loosely bundled fibers not more than 100 μm apart and aperture spaces ranging from 150 to 200 μm wide, with pH=6.5-7.5. Pieces of sponge (10 ×10×5 mm^3^) and their ultrastructure were imaged by scanning electron microscopy (Figure 10).

### Preparation of ginsenoside Rg1

**Figure 11:**
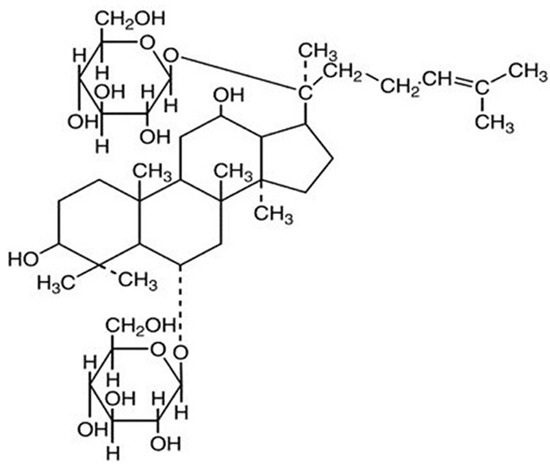
The chemical structure of ginsenoside Rg1 (C_42_H_72_O_14_)

Ginsenoside Rg1 (protopanaxatriol extract monomer; Sigma) was dissolved in pyridine and acetone (both 100μg/mL, final concentration). Its chemical structure is presented in Figure 11.

### Platelet-rich fibrin preparation

**Figure 12:**
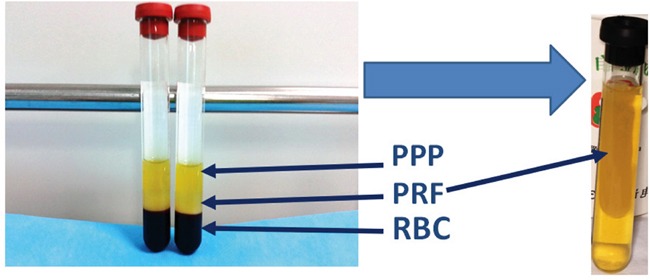
Isolation of PRF clots from venous whole blood after centrifugation: **(A)** Preparation tube following one step centrifugation; the red blood cell elements were in the lower phase, the middle phase containing PRF clots were transferred into a new tube, the upper phase was platelet-poor plasma (PPP)

PRF was prepared as previously reported [18]. Briefly, a patient's blood sample was taken from the median cubital vein, transferred into a 10 mL glass tube without anticoagulation, and centrifuged at 2700 rpm for 12 min. The fibrin clot migrated to the middle of the tube and was easily separated from the red corpuscles at the bottom. After compression with sterile dry gauze, the fluids trapped in the fibrin matrix were driven out and the clot became a very resistant autologous fibrin membrane (Figure 12), which was then cut into pieces for the following experiments.

### Isolation, expansion, and multilineage differentiation identification of HBASCs

HBASCs were isolated from spare fat tissue taken from 6 female reduction mammoplasty and breast prolapse repair patients. HBASCs, after 3^rd^ passage, were used for identification of adipogenic, osteogenic, and chondrogenic differentiation capacity. The detailed experimental method was described in our previous study [19–20].

### Ad-GFP labeling of HBASCs

HBASCs were labeled with green fluorescent protein (GFP) using monster green fluorescent protein vector (Promega, Madison, WI, USA). Ad-GFP was added along with 200 μL of serum-free medium, and the flask was shaken gently every 15 min for 2 h. HBASCs were transduced with the replication-defective recombinant adenovirus at multiplicities of infection (MOI) in the range of 0–200 units. After incubation with Ad-GFP for 2 h, culture medium containing 2.5% FBS was added into the flask. Transduction efficiency was determined by flow cytometry after 48 h. The optimal MOI for transduction of HBASCs was used in the following steps. HBASCs were directly analyzed and green autofluorescence detected by inverted fluorescence microscopy (Leica, Germany). Three days later, the 3^rd^ passage, of HBASCs were collected and employed in this study.

### Effect of ginsenoside Rg1/PRF on HBASCs-scaffold proliferation *in vitro*

For cell proliferation assays, HBASCs at 3^rd^ passage, were cultured in 96-well plates at 10^4^ cells per well with growth culture medium (24 wells for each group). Twenty-four hours later, cells were treated with basal culture media (BCM, group A, control), BCM plus 10 μg/mL Rg1 (group B), BCM plus 10 mg/mL PRF (group C), and BCM plus both Rg1& PRF (group D) for up to 9 days *in vitro*. HBASC proliferation was determined by CCK-8 (Kumamoto, Japan) and measured by microplate reader scanning (ELx800, BioTek) at 450 nm as previously described [21].

### Cell seeding on the scaffolds

The collagen type I sponge scaffolds (COL-S, 10 mm length×10 mm width×5 mm thickness) were put into the 24-well culture plates and divided into 4 groups (6 wells for each group), one piece collagen sponge in each well. Subsequently, HBASCs at 3^rd^ passage, were cultured in 24-well plates at 10^5^ cells per well in growth culture medium (24 wells for each group). Twenty-four hours later, cells were treated with basal culture media (BCM, group A, control), BCM plus 10 μg/mL Rg1 (group B), BCM plus 10 mg/mL PRF (group C), and BCM plus both Rg1& PRF (group D) for up to 7 days *in vitro*. Media was replaced every 2 days.

### Scanning electron microscopy and contrast phase microscopy

After 7 days co-culture of HBASCs and collagen, the morphology of cells growing on the scaffold was evaluated using scanning electron microscopy (SEM, Phenom ProX, Netherlands) images taken on SEM-FE MIRA II LMU (TESCAN, Brno-Kohoutovice, Czech Republic). The samples were gold/palladium coated and analyzed with ImageJ software. The scaffolds were imaged on ESEM XL30 scanning electron microscope (Philips, Eindhoven, Netherlands). Before gold coating, samples were dehydrated and fixed by 2.5% glutaral then washed in PBS, 50, 70, 80, 95, and 100% EtOH, and mixtures of EtOH and Hexamethyldisilazane (HMDS) (Sigma, Aldrich), as well as 100% HMDS solution. Before scanning electron microscopy, we observed the HBASCs using phase contrast microscopy.

### Experimental groups and HBASCs transplantation *in vivo*

Twenty nude mice (average weight 20.0g±4.0g) served as transplantation models. HBASCs were induced in adipogenic differentiation inductive media for one week before implantation. The collagen scaffold-loaded cell suspensions were injected by 16-gauge needles into each mouse subcutaneously at 4 spots (4 groups) which containing 0.5 ml of 1× 10^7^ /ml GFP-labeled HBASCs suspensions (Control, Group A), 0.5 ml of 1× 10^7^ /ml GFP-labeled HBASCs suspensions plus 10 μg/mL Rg1 (Group B), 0.5 ml of 1× 10^7^ /ml GFP-labeled HBASCs suspensions plus 10 mg/mL PRF (Group C), or 0.5 ml of 1× 10^7^ /ml GFP-labeled HBASCs suspensions plus both Rg1& PRF (Group D). Each spot randomly received one of these four cells-scaffold combinations. The mice were fed routinely after operation.

### Tissue wet weight measurement and histopathological examination of transplants

After 3 months, all transplants were excised and weighed on an electronic balance to determine their wet weight. Subsequently, the formalin-maintained transplants were embedded in paraffin. Tissue sections from the center of the dissected regenerative tissue biopsy were stained with hematoxylin-eosin using standard procedures and examined under a light microscope. Neovascularization was assessed by counting the micro vessels in 10 fields of each HE-stained slide (20× magnification; performed by two blinded reviewers).

### Fluorescence microscopy and quantitative measurement of adipogenesis

Transplant samples were embedded, cut into 5-mm-thick slices, and observed under a fluorescence microscope. Sections were stained with Oil Red O followed by washing twice with PBS. The lipids were extracted from the cells by 100% isopropanol and gentle shaking for 5 min. Lipid concentration was measured based on 510 nm absorbance. The lipid quantity and adipocyte density for each sample was double-blindly measured in 6 different visual fields under the same magnification.

### Real-time quantitative PCR analysis (qPCR)

Total RNA was extracted from each group by the TRIzol method following the manufacturer's protocol (Invitrogen, USA). First-strand cDNA was synthesized from 1μg RNA with viral reverse transcriptase (TaKaRa, Japan), and used for quantitative real-time PCR. Expression levels of PPARγ, HIF-1α, and VEGF were quantified with an ABI 7300 real-time PCR system (Applied Biosystems, USA) and SYBR green PCR reaction mix (TaKaRa, Japan). Primers for each gene are listed in Table 2. The program used was 95°C for 5 minutes, 40 cycles of 95°C for 15 seconds, annealing temperature for 1 minute, and 72°C for 30 s. Melting analysis and agarose gel electrophoresis were performed to confirm the specificity of the PCR products. The relative expression levels of genes were analyzed using the 2^−ΔΔCt^ method by normalizing with GAPDH housekeeping gene expression, and presented as fold increase relative to the control group.

**Table 2:** Primer sequences

Gene name (human)	Forward primer sequence (5′ to 3′)	Reverse primer sequence (5′ to 3′)
PPARγ	GGCCGTCTATGTGGGTGTCTGG	TGGCCCTTGGAGTGTGACAG
HIF-1α	AAGTCTAGGGATGCAGCAC	CAAGATCACCAGCATCTAG
VEGF	ATGGCAGAAGGAGGAGGG	CGAAACGCTGAGGGAGGCT
GAPDH	AGAAGGCTGGGGCTCATTTG	AGGGGCCATCCACAGTCTTC

### Protein extraction and western blotting

Ten samples collected from the neogenetic adipose tissue in 4 groups were harvested for Western Blot analysis and the whole cell extracts were obtained. Briefly, cell pellets were sonicated in extraction buffer. Extracts were quantified using the Bio-Rad DC protein assay kit (BioRad, Hercules, Calif.). Equal amounts of protein were resolved on 4%–12% SDS–PAGE and transferred to PVDF membranes (Millipore, Bedford, Massachusetts, USA). Membranes were blocked with blocking solution (Pierce, Rockford, Illinois, USA). Primary antibodies used were: anti-human PPARγ, anti-human HIF-1α, and anti-human VEGF (all from Abcam, London, UK). Horseradish-peroxidase-conjugated secondary antibody and enhanced chemiluminescence substrate (Supersignal West Dura Detection System; Pierce) were used for primary antibody detection.

### Statistical analysis

Data are shown as mean±SD. Since each mouse was randomly injected with the four groups at 4 spots, we performed analysis of variance to determine whether the means of all groups were equal. This approach takes into account within-subject and between-subject variation. Furthermore, if the analysis of variance of the four means revealed statistically significant differences, multiple comparisons of the means of any two groups were made using the paired T-test. Differences in means were regarded as statistically significant if a two-tail p value was less than 0.05. All data were analyzed by using SPSS for Windows 16.0 version (Chicago, IL, USA).

## CONCLUSION

In this study, we found that HBASCs-attached scaffolds treated with G-Rg1 or PRF increase neovascularization and enhance tissue engineering adipogenesis. Moreover, combined application of both factors augments this effect. There is excellent biocompatibility between HBASCs and 3D porous collagen type I scaffolds which is improved by ginsenoside Rg1 and platelet rich fibrin for soft tissue regeneration.
